# Update on Genetic Basis of Brugada Syndrome: Monogenic, Polygenic or Oligogenic?

**DOI:** 10.3390/ijms21197155

**Published:** 2020-09-28

**Authors:** Oscar Campuzano, Georgia Sarquella-Brugada, Sergi Cesar, Elena Arbelo, Josep Brugada, Ramon Brugada

**Affiliations:** 1 Cardiovascular Genetics Centre, University of Girona-IDIBGI, 17190 Girona, Spain; 2 Medical Science Department, School of Medicine, University of Girona, 17003 Girona, Spain; georgia@brugada.org; 3 Centro Investigación Biomédica en Red: Enfermedades Cardiovasculares (CIBERCV), 28029 Madrid, Spain; elenaarbelo@secardiologia.es (E.A.); jbrugada@clinic.cat (J.B.); 4 Arrhythmia Unit, Hospital Sant Joan de Déu, University of Barcelona, 08950 Barcelona, Spain; sergi.cesar@gmail.com; 5 Arrhythmia Section, Cardiovascular Institute, Hospital Clinic, University of Barcelona, 08036 Barcelona, Spain; 6 Familial Cardiomyopathies Unit, Hospital Josep Trueta de Girona, 17007 Girona, Spain

**Keywords:** Brugada syndrome, arrhythmias, sudden cardiac death, genetics

## Abstract

Brugada syndrome is a rare inherited arrhythmogenic disease leading to ventricular fibrillation and high risk of sudden death. In 1998, this syndrome was linked with a genetic variant with an autosomal dominant pattern of inheritance. To date, rare variants identified in more than 40 genes have been potentially associated with this disease. Variants in regulatory regions, combinations of common variants and other genetic alterations are also proposed as potential origins of Brugada syndrome, suggesting a polygenic or oligogenic inheritance pattern. However, most of these genetic alterations remain of questionable causality; indeed, rare pathogenic variants in the *SCN5A* gene are the only established cause of Brugada syndrome. Comprehensive analysis of all reported genetic alterations identified the origin of disease in no more than 40% of diagnosed cases. Therefore, identifying the cause of this rare arrhythmogenic disease in the many families without a genetic diagnosis is a major current challenge in Brugada syndrome. Additional challenges are interpretation/classification of variants and translation of genetic data into clinical practice. Further studies focused on unraveling the pathophysiological mechanisms underlying the disease are needed. Here we provide an update on the genetic basis of Brugada syndrome.

## 1. Introduction

Brugada syndrome (BrS) was first described in eight individuals who were resuscitated from sudden cardiac death (SCD) due to ventricular fibrillation (VF) in a structurally normal heart [1]. BrS is diagnosed by a “type 1” electrocardiogram (ECG) pattern characterized by ST segment elevation and a coved-type morphology ≥ 0.2 mV in one of the right precordial leads (V1 and V2) positioned in the second, third, or fourth intercostal space; this pattern can occur in the presence or absence of intravenous administration of Class I antiarrhythmic drugs such as ajmaline, flecainide, and procainamide [2]. Importantly, SCD is often the first symptom of BrS, predominantly in adult males at night or during rest. Early identification of at-risk individuals is crucial to prevent lethal episodes, underscoring the need for improved diagnostic tools [3]. Despite initial recognition of BrS as a familial disease, the first genetic alteration supporting its inheritance pattern was not reported until 1998 [4]. Indeed, efforts focused on identifying the genetic cause of BrS are important for detecting asymptomatic genetic carriers at risk for SCD and for stratifying the risk of future arrhythmic events [5]. Families diagnosed with BrS show incomplete penetrance and variable expressivity, and genetic variants resulting in or predisposing to BrS could be misinterpreted [6]. The absence of a genotype–phenotype correlation in families affected by BrS underscores the complexity of this arrhythmogenic entity and suggests that the occurrence and prognosis of BrS is most likely influenced by a combination of multiple genetic alterations and environmental factors, involving a fine balance of ion currents [7,8].

## 2. Genetic Alterations

### 2.1. Genetic Causality

The first genetic alteration associated with BrS was reported in 1998 [4] in the sodium channel protein type V subunit α gene (*SCN5A*), encoding the alpha subunit of the voltage-gated NaV1.5 cardiac sodium channel. NaV1.5 regulates rapid sodium current in myocytes. Over 500 rare alterations in more than 40 genes have since been reported as potentially associated with BrS. These genes primarily encode sodium, potassium, and calcium channels or proteins associated with function of these ion channels [9]. Identification of other genes potentially associated with BrS has enabled genetic testing in the clinical workup. However, comprehensive analysis of all potential BrS-associated genes identified a disease-causing alteration in no more than 40% of diagnosed cases, leaving most families with an unknown genetic origin of the disease [10].

Currently, *SCN5A* is the main gene associated with BrS and nearly 30% of diagnosed cases are attributed to one of the more than 350 rare variants identified in this gene [3]. Copy number variations (CNVs) are additional rare alterations potentially causative of BrS. Of fewer than 10 CNVs potentially associated with BrS to date, all are located in the *SCN5A* gene [11,12,13]. Recently, patients affected by BrS carrying a gross deletion of the *GSTM3* gene showed higher rates of syncope and SCD compared to those without, suggesting a novel potential genetic modifier/risk predictor for the development of BrS. However, further studies should be done to clarify the role of this CNV in BrS [14]. Then, focusing only in *SCN5A*, CNVs appear to underlie no more than 1% of diagnosed BrS cases. Consequently, current guidelines recommend analyzing only *SCN5A* in suspected BrS cases as the most cost-effective approach [2]. This guideline was recently supported by an exhaustive genetic analysis of families affected by BrS, showing only *SCN5A* to be a definitive deleterious gene. However, the percentage of deleterious variants in *SCN5A* may be overestimated because many rare variants previously classified as pathogenic/likely pathogenic are now categorized as being of ambiguous significance [15], according to American College of Medical Genetics and Genomics and Association for Molecular Pathology (ACMG/AMP) guidelines [16]. Recent reevaluations of all potentially BrS-associated rare variants in *SCN5A* classified only 37–47% as deleterious [17,18].

**Figure 1:**
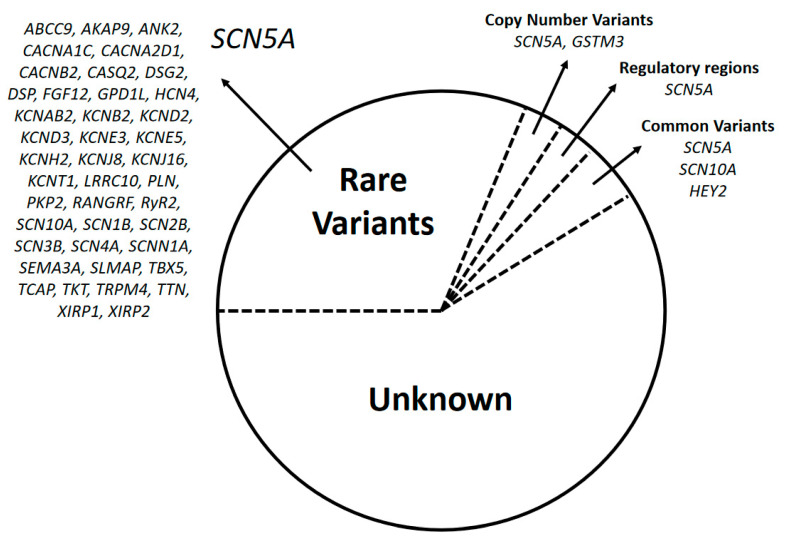
Genetics of Brugada syndrome (BrS). After a comprehensive genetic analysis, almost 60% of patients remain without a genetic diagnosis. No more than 35% of cases are due to rare variants; of them, almost 30% are located in the *SCN5A* gene. Other alterations such as copy number variants, variants in regulatory regions and common variants are responsible altogether for less than 10% of BrS cases.

Almost 150 additional variants in other genes are proposed to be causative of BrS, but together these explain no more than 10% of cases and they are thus considered minor genes (*ABCC9, AKAP9, ANK2, CACNA1C, CACNA2D1, CACNB2, CASQ2, DSG2, DSP, FGF12, GPD1L, GSTM3, HCN4, HEY2, KCNAB2, KCNB2, KCND2, KCND3, KCNE3, KCNE5, KCNH2, KCNJ8, KCNJ16, KCNT1, LRRC10, PLN, PKP2, RANGRF, RyR2, SCN10A, SCN1B, SCN2B, SCN3B, SCN4A, SCNN1A, SEMA3A, SLMAP, TBX5, TCAP, TKT, TRPM4, TTN, XIRP1,* and *XIRP2*) (Figure 1). Most of these variants are rare, but genome-wide association studies (GWAS) found that common variants in *SCN5A, SCN10A,* and *HEY2* might modulate gene expression and susceptibility to BrS in individuals of European and Japanese ancestry [19,20]. Recently, common variants near *SCN5A* and *HEY2* were associated with BrS in the Thai population, confirming the trans-ethnicity of these two major BrS loci [21]. However, another recent study validated only 1/4 of previously identified common variants in patients affected by BrS from Taiwan [22]. Additionally, due to a lack of common variants, the differing allele frequencies in patients affected by BrS with different ethnic backgrounds should also be taken into consideration during classification and clinical translation [22].

In 2018, mutations in mitochondrial tRNA genes that may affect the translation of critical proteins of the respiratory chain were proposed as potentially leading to BrS [23]. Additionally, alterations in the core promoter region and the transcriptional regulatory region of *SCN5A* were recently described in BrS, although they explain only 1% of cases [24,25,26,27]. This finding highlights the impact of non-coding region variants, which are binding targets for miRNAs, in altering miRNA-target cross-talk. Rare compound alterations suggest that single variants in *SCN5A* are not sufficient to cause BrS [28]. Genetic background may therefore be a powerful modulator of disease expression [29], and compound variants may be associated with more severe BrS phenotypes [30], although nothing conclusive has been reported so far. Further genotype–phenotype studies in large cohorts supported by functional analysis (in vitro/in vivo) are needed to clarify the true pathogenic roles of the various genetic alterations in BrS.

### 2.2. Genetic Modulation of Phenotype

Recently, common variants in *SCN5A* were reported to play a regulatory function similar to that of rare deleterious variants in the same gene and, therefore, modulate phenotype expression in BrS [31]. Some of these polymorphisms decrease *SCN5A* promoter methylation and increase *SCN5A* expression in cardiac tissue, leading to the BrS phenotype [32]. MicroRNA expression may also be a genetic modifier of many *SCN5A* rare variants causal for BrS [33], and has been associated with a high risk of subsequent cardiac arrhythmias [34]. MicroRNA binding affinity does not co-segregate with BrS in affected family members, supporting an additive contribution to BrS onset rather than a unique causative link, and opens a new pathway that may cause and/or modulate BrS [35]. Finally, the BrS phenotype can be induced by drugs (www.brugadadrugs.org) and various environmental factors such as hormones, alcohol, or fever [36]. Fever is especially important in the pediatric population. However, a recent study suggests that recording of fever-ECGs may not be mandatory in pediatric patients from families affected by BrS without a disease variant in *SCN5A* because their risk for a febrile Brugada type-1 ECG and malignant arrhythmic event is low [37].

The function of NaV1.5 may be affected by rare and/or common variants in *SCN5A* and other genes encoding proteins that interact with sodium channels. NaV1.5 may modulate its function through different mechanisms (decreased channel expression, channel dysfunction, or alteration in gating properties) leading to VF and SCD even in asymptomatic individuals. Thus, even when no genetic variant has been identified, the drugs and dangerous situations known to induce the BrS phenotype should be avoided in families with diagnosed or suspected BrS [3].

Genotype–phenotype studies have established that more than 95% of rare alterations identified in patients affected by BrS follow an autosomal dominant pattern of inheritance. However, autosomal recessive [38] and X-linked [39] inheritance have also been identified in a few cases. Further, a specific mitochondrial DNA allelic combination may be associated with increased phenotype severity [40]. Both common and rare variants are increasingly associated with BrS. Thus, accumulation of genetic variants of diverse allele frequencies and effect sizes may modulate disease susceptibility, suggesting that the currently accepted Mendelian inheritance should be replaced with a polygenic or oligogenic model [36,41]. This is supported by the finding that sodium channel blockers do not provoke the characteristic BrS pattern in ECGs in healthy individuals or in all carriers of *SCN5A* rare variants [42]. Additional genotype–phenotype studies should be performed in large BrS cohorts to facilitate identification of a definite causative pattern of inheritance in families affected by BrS.

## 3. Genetic Interpretation/Classification

In BrS, the first step before interpretation of genetic data should be a definitive clinical diagnosis. The characteristic ST-segment elevation in the ECG may occur due to non-BrS-related causes, such as RV ischemia, acute pulmonary embolism, or mechanical compression of the RV outflow tract, mimicking BrS and called Brugada ECG phenocopy [43]. Because it is an inherited disease, clinical assessment and comprehensive genotype–phenotype analysis of all available relatives may help clinicians to identify BrS in uncertain cases. Both incomplete penetrance and variable expressivity should be considered in pedigree performance [6]. When a definitive diagnosis of BrS is obtained, interpretation of genetic data should follow current ACMG/AMP guidelines [16]. These guidelines recommend classifying pathogenicity by global frequencies, previous classification in other cases, and functional studies. BrS is a rare disease with an estimated prevalence from 1/5000 to 1/2000 [44]. The prevalence is higher in Southeast Asia, and thus, racial/ethnic origin of each BrS patient should be considered. Minor allele frequencies (MAF) less than 0.02–0.05% can be potentially causative depending on ethnicity. Rare variants can be found in about 2% of global population and in 5% of non-white populations [45]. There is a 10-to-1 genetic signal-to-noise ratio that indicates a 10% risk of false positives in possibly damaging rare *SCN5A* variants [46]. However, this percentage may be overestimated because the prevalence of asymptomatic patients affected by BrS in the general population is unknown. Therefore, due to the rarity of the disease, a real potentially causative variant should have a MAF under 0.01% [47]. Indeed, recent reevaluations of all potentially BrS-associated variants in *SCN5A* concluded that only 37–47% of rare variants previously considered to be potentially damaging should remain classified as deleterious [17,18]. Therefore, available genetic data on each variant of interest should be exhaustively analyzed periodically in order to include new data in interpretation/classification before clinical translation. Despite this fact, no precise time of reevaluation has been stated to date.

Functional evidence should incorporate in silico, in vitro, and in vivo analyses, although all models have limitations [48]. In silico analyses use mathematical tools to support defining variant pathogenicity. Existing programs can produce discordant attributions due to the use of different algorithms. Therefore, in silico prediction may be uncertain or inaccurate in more than 20% of cases [49]. In silico predictors should therefore be combined with other functional analyses for variant pathogenicity [50]. Cellular models can reveal the biophysical consequences of wild type and BrS-associated mutant ion channel expression. A combined technical approach of patch clamp and heterologous expression systems was long the only experimental model allowing investigation of the molecular pathways involved in BrS [48]. Recent development of induced pluripotent stem cell-derived cardiomyocyte (iPS-CM) technology creates an opportunity to study cardiomyocytes derived from patients and healthy individuals. Several studies have used iPS-CM derived from patients affected by BrS to explore novel therapeutic tools and to study the basic mechanisms of this arrhythmogenic disease [51,52]. Compared to single-cell approaches, the iPS-CM approach can more accurately identify the pathophysiological mechanisms involved in BrS [53]. Finally, in vivo models including transgenic mice (knock-out and knock-in), transgenic pigs (limited studies), and canine heart preparations (using arterially perfused wedges of left or right ventricles rather than a whole heart) can simulate BrS phenotypes but are not commonly used due to their costliness [48].

Using available clinical and functional data as well as other information included in ACMG/AMP guidelines, each variant is assigned a final score and is consequently classified as pathogenic (P), likely pathogenic (LP), variant of uncertain significance (VUS), likely benign (LB), and benign (B). This classification system was designed to have general applicability, and therefore further studies of specific diseases and genes are necessary to enable robust clinical translation. Consequently, several updates were recently proposed to improve the accuracy of this classification system [47,54,55]. Importantly, all classifications are performed according to the data available at the time of interpretation. Therefore, predictions regarding BrS are not immutable, and classifications of analyzed variants should be revised periodically as new data can be available [56,57]. New data included in the final classification may modify the role of a variant, with potential clinical implications. Despite this key role, as mentioned above, no precise time of reevaluation has been stated to date.

Correlation with patient clinical information as well as familial segregation are crucial to performing a real interpretation. For example, in a patient diagnosed with BrS, a rare variant may be classified as LP according to the available genetic data, but if any relatives also clinically diagnosed with BrS do not carry the same variant, this implies that the variant is not the cause of BrS in the family. However, the rare variant could be associated with phenotype modification. Thus, all available data on a BrS-related variant should be collected and comprehensively interpreted to obtain a proper classification. However, from our point of view, familial segregation is mandatory before associating rare variants with disease susceptibility. This would facilitate translation into clinical practice.

## 4. Clinical Translation

It is important that genetic interpretation and clinical translation be performed together. Clinical data are crucial to clarifying the role of a genetic variant and translation of genetic data into clinical practice should be performed after comprehensive genetic interpretation/classification. In a family diagnosed with BrS, genetic interpretation and clinical translation should be performed with the consensus of a group of experts including cardiologists, pediatricians, pediatric cardiologists, geneticists and genetic counselors. Because SCD can be the first manifestation of BrS, forensic and cardiac pathologists should also be included in the team of experts. We believe that creating a group of experts is the best approach to personalized management, including diagnosis, treatment, and prevention of lethal arrhythmias in individuals at risk.

*SCN5A* is the only gene with conclusive association with BrS to date. In patients with a rare variant in the *SCN5A* gene, prolonged P-wave, PR, or QRS duration are frequently observed [58]. Additionally, the combination of an *SCN5A* rare variant with a history of SCD in a first-degree relative under 35 years old seems to be predictive of arrhythmic events [59,60]. Recently, a study showed that symptomatic patients with variants in *SCN5A* were at higher risk of arrhythmic events than symptomatic *SCN5A*-variant-negative patients [61]. Despite these recent advances, genetic data do not currently influence the prognosis or treatment of BrS [2].

One of the main difficulties in patients affected by BrS is determining if a rare variant is a real pathogenic alteration responsible for the disease. Following clinical guidelines, only variants classified as P and LP should be strongly considered as damaging in BrS [2]. Currently, the large number of items included in ACMG/AMP classification confers accuracy but also stringency, thus most rare variants identified remain VUS [56]. Therefore, current data do not allow conclusive classification and further studies are needed to clarify the roles of VUS in BrS. We believe that VUS in patients affected by BrS should not be discarded due to limitations in published data. However, only definitive genetic data should be translated to families to avoid confusion or harm [62]. In our point of view after more than 25 years of experience in the field, families should receive a complete explanation of the genetic data obtained, helping to clarify the potential role of each rare variant, although this should not serve as the basis for clinical decisions. Finally, variants classified as LB and B can be discarded as the cause of BrS mainly due to their high prevalence in the global population. However, a variant classified as LB in patients from Southeast Asia should be widely revised because of the higher prevalence of BrS. Additionally, variants classified as LB and B may be phenotype modifiers in BrS and should be included in the patient’s genetic report [32,63,64,65].

## 5. Conclusions

BrS is a rare inherited arrhythmia associated with risk of sudden death in the young adult population. To date, only rare variants in *SCN5A* have been definitively associated with BrS, and following an autosomal dominant mode of transmission. However, incomplete penetrance and variable expressivity suggest a complex mode of inheritance most likely controlled by a combination of multiple genetic factors influenced by the environment, rather than a single rare variant. The lack of conclusive data concerning these additional genetic alterations means BrS remains classified as a monogenic disease. Additionally, more than 60% of families remain without a genetic diagnosis after exhaustive genetic analysis. The lack of functional models impedes proper interpretation of genetic alterations, and most remain classified as being of ambiguous significance. Therefore, extreme caution should be taken before translation of genetic data into clinical practice. There is a need for the better understanding of the genetic architecture of BrS before applying genetics as a diagnostic and risk stratification tool among patients.

## References

[1] Brugada P., Brugada J. (1992). Right bundle branch block, persistent ST segment elevation and sudden cardiac death: A distinct clinical and electrocardiographic syndrome. A multicenter report. J. Am. Coll. Cardiol..

[2] Priori S.G., Blomstrom-Lundqvist C. (2015). 2015 European Society of Cardiology Guidelines for the management of patients with ventricular arrhythmias and the prevention of sudden cardiac death summarized by co-chairs. Eur. Heart J..

[3] Brugada J., Campuzano O., Arbelo E., Sarquella-Brugada G., Brugada R. (2018). Present Status of Brugada Syndrome: JACC State-of-the-Art Review. J. Am. Coll. Cardiol..

[4] Chen Q., Kirsch G.E., Zhang D., Brugada R., Brugada J., Brugada P., Potenza D., Moya A., Borggrefe M., Breithardt G. (1998). Genetic basis and molecular mechanism for idiopathic ventricular fibrillation. Nature.

[5] Garcia J., Tahiliani J., Johnson N.M., Aguilar S., Beltran D., Daly A., Decker E., Haverfield E., Herrera B., Murillo L. (2016). Clinical Genetic Testing for the Cardiomyopathies and Arrhythmias: A Systematic Framework for Establishing Clinical Validity and Addressing Genotypic and Phenotypic Heterogeneity. Front. Cardiovasc. Med..

[6] Coll M., Perez-Serra A., Mates J., Del Olmo B., Puigmule M., Fernandez-Falgueras A., Iglesias A., Pico F., Lopez L., Brugada R. (2017). Incomplete Penetrance and Variable Expressivity: Hallmarks in Channelopathies Associated with Sudden Cardiac Death. Biology.

[7] Kline J., Costantini O. (2019). Inherited Cardiac Arrhythmias and Channelopathies. Med. Clin. N. Am..

[8] Skinner J.R., Winbo A., Abrams D., Vohra J., Wilde A.A. (2019). Channelopathies That Lead to Sudden Cardiac Death: Clinical and Genetic Aspects. Heart Lung Circ..

[9] Campuzano O., Sarquella-Brugada G., Fernandez-Falgueras A., Cesar S., Coll M., Mates J., Arbelo E., Perez-Serra A., Del Olmo B., Jorda P. (2019). Genetic interpretation and clinical translation of minor genes related to Brugada syndrome. Hum. Mutat..

[10] Coppola G., Corrado E., Curnis A., Maglia G., Oriente D., Mignano A., Brugada P. (2019). Update on Brugada Syndrome 2019. Curr. Probl. Cardiol..

[11] Eastaugh L.J., James P.A., Phelan D.G., Davis A.M. (2011). Brugada syndrome caused by a large deletion in SCN5A only detected by multiplex ligation-dependent probe amplification. J. Cardiovasc. Electrophysiol..

[12] Mademont-Soler I., Pinsach-Abuin M.L., Riuro H., Mates J., Perez-Serra A., Coll M., Porres J.M., Del Olmo B., Iglesias A., Selga E. (2016). Large Genomic Imbalances in Brugada Syndrome. PLoS ONE.

[13] Sonoda K., Ohno S., Ozawa J., Hayano M., Hattori T., Kobori A., Yahata M., Aburadani I., Watanabe S., Matsumoto Y. (2018). Copy number variations of SCN5A in Brugada syndrome. Heart Rhythm.

[14] Juang J.J., Binda A., Lee S.J., Hwang J.J., Chen W.J., Liu Y.B., Lin L.Y., Yu C.C., Ho L.T., Huang H.C. (2020). GSTM3 variant is a novel genetic modifier in Brugada syndrome, a disease with risk of sudden cardiac death. EBioMedicine.

[15] Hosseini S.M., Kim R., Udupa S., Costain G., Jobling R., Liston E., Jamal S.M., Szybowska M., Morel C.F., Bowdin S. (2018). Reappraisal of Reported Genes for Sudden Arrhythmic Death: An Evidence-Based Evaluation of Gene Validity for Brugada Syndrome. Circulation.

[16] Richards S., Aziz N., Bale S., Bick D., Das S., Gastier-Foster J., Grody W.W., Hegde M., Lyon E., Spector E. (2015). Standards and guidelines for the interpretation of sequence variants: A joint consensus recommendation of the American College of Medical Genetics and Genomics and the Association for Molecular Pathology. Genet. Med..

[17] Denham N.C., Pearman C.M., Ding W.Y., Waktare J., Gupta D., Snowdon R., Hall M., Cooper R., Modi S., Todd D. (2018). Systematic Re-evaluation of SCN5A Variants Associated with Brugada Syndrome. J. Cardiovasc. Electrophysiol..

[18] Kroncke B.M., Glazer A.M., Smith D.K., Blume J.D., Roden D.M. (2018). SCN5A (NaV1.5) Variant Functional Perturbation and Clinical Presentation: Variants of a Certain Significance. Circ. Genom. Precis. Med..

[19] Bezzina C.R., Barc J., Mizusawa Y., Remme C.A., Gourraud J.B., Simonet F., Verkerk A.O., Schwartz P.J., Crotti L., Dagradi F. (2013). Common variants at SCN5A-SCN10A and HEY2 are associated with Brugada syndrome, a rare disease with high risk of sudden cardiac death. Nat. Genet..

[20] van den Boogaard M., Smemo S., Burnicka-Turek O., Arnolds D.E., van de Werken H.J., Klous P., McKean D., Muehlschlegel J.D., Moosmann J., Toka O. (2014). A common genetic variant within SCN10A modulates cardiac SCN5A expression. J. Clin. Investig..

[21] Makarawate P., Glinge C., Khongphatthanayothin A., Walsh R., Mauleekoonphairoj J., Amnueypol M., Prechawat S., Wongcharoen W., Krittayaphong R., Anannab A. (2020). Common and rare susceptibility genetic variants predisposing to Brugada Syndrome in Thailand. Heart Rhythm.

[22] Juang J.J., Liu Y.B., Chen C.J., Yu Q.Y., Chattopadhyay A., Lin L.Y., Chen W.J., Yu C.C., Huang H.C., Ho L.T. (2020). Validation and Disease Risk Assessment of Previously Reported Genome-Wide Genetic Variants Associated with Brugada Syndrome: SADS-TW BrS Registry. Circ. Genom. Precis. Med..

[23] Tafti M.F., Khatami M., Rezaei S., Heidari M.M., Hadadzadeh M. (2018). Novel and heteroplasmic mutations in mitochondrial tRNA genes in Brugada syndrome. Cardiol. J..

[24] Yagihara N., Watanabe H., Barnett P., Duboscq-Bidot L., Thomas A.C., Yang P., Ohno S., Hasegawa K., Kuwano R., Chatel S. (2016). Variants in the SCN5A Promoter Associated With Various Arrhythmia Phenotypes. J. Am. Heart Assoc..

[25] Beltran-Alvarez P., Espejo A., Schmauder R., Beltran C., Mrowka R., Linke T., Batlle M., Perez-Villa F., Perez G.J., Scornik F.S. (2013). Protein arginine methyl transferases-3 and -5 increase cell surface expression of cardiac sodium channel. FEBS Lett..

[26] Beltran-Alvarez P., Tarradas A., Chiva C., Perez-Serra A., Batlle M., Perez-Villa F., Schulte U., Sabido E., Brugada R., Pagans S. (2014). Identification of N-terminal protein acetylation and arginine methylation of the voltage-gated sodium channel in end-stage heart failure human heart. J. Mol. Cell Cardiol..

[27] Tarradas A., Pinsach-Abuin M.L., Mackintosh C., Llora-Batlle O., Perez-Serra A., Batlle M., Perez-Villa F., Zimmer T., Garcia-Bassets I., Brugada R. (2017). Transcriptional regulation of the sodium channel gene (SCN5A) by GATA4 in human heart. J. Mol. Cell Cardiol..

[28] Cordeiro J.M., Barajas-Martinez H., Hong K., Burashnikov E., Pfeiffer R., Orsino A.M., Wu Y.S., Hu D., Brugada J., Brugada P. (2006). Compound heterozygous mutations P336L and I1660V in the human cardiac sodium channel associated with the Brugada syndrome. Circulation.

[29] Probst V., Wilde A.A., Barc J., Sacher F., Babuty D., Mabo P., Mansourati J., Le Scouarnec S., Kyndt F., Le Caignec C. (2009). SCN5A mutations and the role of genetic background in the pathophysiology of Brugada syndrome. Circ. Cardiovasc. Genet..

[30] Sacilotto L., Epifanio H.B., Darrieux F.C., Wulkan F., Oliveira T.G., Hachul D.T., Pereira A.D., Scanavacca M.I. (2017). Compound Heterozygous SCN5A Mutations in a Toddler—Are they Associated with a More Severe Phenotype?. Arq. Bras. Cardiol..

[31] Makarawate P., Chaosuwannakit N., Vannaprasaht S., Sahasthas D., Koo S.H., Lee E.J.D., Tassaneeyakul W., Barajas-Martinez H., Hu D., Sawanyawisuth K. (2017). SCN5A Genetic Polymorphisms Associated With Increased Defibrillator Shocks in Brugada Syndrome. J. Am. Heart Assoc..

[32] Matsumura H., Nakano Y., Ochi H., Onohara Y., Sairaku A., Tokuyama T., Tomomori S., Motoda C., Amioka M., Hironobe N. (2017). H558R, a common SCN5A polymorphism, modifies the clinical phenotype of Brugada syndrome by modulating DNA methylation of SCN5A promoters. J. Biomed. Sci..

[33] Wilde A.A., Brugada R. (2011). Phenotypical manifestations of mutations in the genes encoding subunits of the cardiac sodium channel. Circ. Res..

[34] Ritchie M.D., Denny J.C., Zuvich R.L., Crawford D.C., Schildcrout J.S., Bastarache L., Ramirez A.H., Mosley J.D., Pulley J.M., Basford M.A. (2013). Genome- and phenome-wide analyses of cardiac conduction identifies markers of arrhythmia risk. Circulation.

[35] Daimi H., Khelil A.H., Neji A., Hamda B.K., Maaoui S., Aranega A., Be Chibani J., Franco D. (2019). Role of SCN5A coding and non-coding sequences in Brugada syndrome onset: What’s behind the scenes?. Biomed. J..

[36] Monasky M.M., Micaglio E., Ciconte G., Pappone C. (2020). Brugada Syndrome: Oligogenic or Mendelian Disease?. Int. J. Mol. Sci..

[37] Peltenburg P.J., Blom N.A., Vink A.S., Kammeraad J.A.E., Breur H., Rammeloo L.A.J., Wilde A.A.M., Clur S.B. (2020). In Children and Adolescents from Brugada Syndrome-Families, Only SCN5A Mutation Carriers Develop a Type-1 ECG Pattern Induced by Fever. Circulation.

[38] Janin A., Bessiere F., Georgescu T., Chanavat V., Chevalier P., Millat G. (2019). TRPM4 mutations to cause autosomal recessive and not autosomal dominant Brugada type 1 syndrome. Eur. J. Med Genet..

[39] David J.P., Lisewski U., Crump S.M., Jepps T.A., Bocksteins E., Wilck N., Lossie J., Roepke T.K., Schmitt N., Abbott G.W. (2019). Deletion in mice of X-linked, Brugada syndrome- and atrial fibrillation-associated Kcne5 augments ventricular KV currents and predisposes to ventricular arrhythmia. FASEB J..

[40] Stocchi L., Polidori E., Potenza L., Rocchi M.B., Calcabrini C., Busacca P., Capalbo M., Potenza D., Amati F., Mango R. (2016). Mutational analysis of mitochondrial DNA in Brugada syndrome. Cardiovasc. Pathol..

[41] Cerrone M., Remme C.A., Tadros R., Bezzina C.R., Delmar M. (2019). Beyond the One Gene-One Disease Paradigm: Complex Genetics and Pleiotropy in Inheritable Cardiac Disorders. Circulation.

[42] Hoogendijk M.G., Opthof T., Postema P.G., Wilde A.A., de Bakker J.M., Coronel R. (2010). The Brugada ECG pattern: A marker of channelopathy, structural heart disease, or neither? Toward a unifying mechanism of the Brugada syndrome. Circ. Arrhythm. Electrophysiol..

[43] Baranchuk A., Nguyen T., Ryu M.H., Femenia F., Zareba W., Wilde A.A., Shimizu W., Brugada P., Perez-Riera A.R. (2012). Brugada phenocopy: New terminology and proposed classification. Ann. Noninvasive Electrocardiol..

[44] Vutthikraivit W., Rattanawong P., Putthapiban P., Sukhumthammarat W., Vathesatogkit P., Ngarmukos T., Thakkinstian A. (2018). Worldwide Prevalence of Brugada Syndrome: A Systematic Review and Meta-Analysis. Acta Cardiol. Sin..

[45] Kapa S., Tester D.J., Salisbury B.A., Harris-Kerr C., Pungliya M.S., Alders M., Wilde A.A., Ackerman M.J. (2009). Genetic testing for long-QT syndrome: Distinguishing pathogenic mutations from benign variants. Circulation.

[46] Ackerman M.J. (2015). Genetic purgatory and the cardiac channelopathies: Exposing the variants of uncertain/unknown significance issue. Heart Rhythm.

[47] Kobayashi Y., Yang S., Nykamp K., Garcia J., Lincoln S.E., Topper S.E. (2017). Pathogenic variant burden in the ExAC database: An empirical approach to evaluating population data for clinical variant interpretation. Genome Med..

[48] Sendfeld F., Selga E., Scornik F.S., Perez G.J., Mills N.L., Brugada R. (2019). Experimental Models of Brugada syndrome. Int. J. Mol. Sci..

[49] Frousios K., Iliopoulos C.S., Schlitt T., Simpson M.A. (2013). Predicting the functional consequences of non-synonymous DNA sequence variants—Evaluation of bioinformatics tools and development of a consensus strategy. Genomics.

[50] Giudicessi J.R., Ackerman M.J. (2013). Genetic testing in heritable cardiac arrhythmia syndromes: Differentiating pathogenic mutations from background genetic noise. Curr. Opin. Cardiol..

[51] Liang P., Sallam K., Wu H., Li Y., Itzhaki I., Garg P., Zhang Y., Vermglinchan V., Lan F., Gu M. (2016). Patient-Specific and Genome-Edited Induced Pluripotent Stem Cell-Derived Cardiomyocytes Elucidate Single-Cell Phenotype of Brugada Syndrome. J. Am. Coll. Cardiol..

[52] Veerman C.C., Mengarelli I., Guan K., Stauske M., Barc J., Tan H.L., Wilde A.A., Verkerk A.O., Bezzina C.R. (2016). hiPSC-derived cardiomyocytes from Brugada Syndrome patients without identified mutations do not exhibit clear cellular electrophysiological abnormalities. Sci. Rep..

[53] Selga E., Sendfeld F., Martinez-Moreno R., Medine C.N., Tura-Ceide O., Wilmut S.I., Perez G.J., Scornik F.S., Brugada R., Mills N.L. (2018). Sodium channel current loss of function in induced pluripotent stem cell-derived cardiomyocytes from a Brugada syndrome patient. J. Mol. Cell Cardiol..

[54] Lek M., Karczewski K.J., Minikel E.V., Samocha K.E., Banks E., Fennell T., O’Donnell-Luria A.H., Ware J.S., Hill A.J., Cummings B.B. (2016). Analysis of protein-coding genetic variation in 60,706 humans. Nature.

[55] Abou Tayoun A.N., Pesaran T., DiStefano M.T., Oza A., Rehm H.L., Biesecker L.G., Harrison S.M. (2018). ClinGen Sequence Variant Interpretation Working G: Recommendations for interpreting the loss of function PVS1 ACMG/AMP variant criterion. Hum. Mutat..

[56] Campuzano O., Sarquella-Brugada G., Fernandez-Falgueras A., Coll M., Iglesias A., Ferrer-Costa C., Cesar S., Arbelo E., Garcia-Alvarez A., Jorda P. (2020). Reanalysis and reclassification of rare genetic variants associated with inherited arrhythmogenic syndromes. EBioMedicine.

[57] Bennett J.S., Bernhardt M., McBride K.L., Reshmi S.C., Zmuda E., Kertesz N.J., Garg V., Fitzgerald-Butt S., Kamp A.N. (2019). Reclassification of Variants of Uncertain Significance in Children with Inherited Arrhythmia Syndromes is Predicted by Clinical Factors. Pediatric Cardiol..

[58] Smits J.P., Eckardt L., Probst V., Bezzina C.R., Schott J.J., Remme C.A., Haverkamp W., Breithardt G., Escande D., Schulze-Bahr E. (2002). Genotype-phenotype relationship in Brugada syndrome: Electrocardiographic features differentiate SCN5A-related patients from non-SCN5A-related patients. J. Am. Coll. Cardiol..

[59] Sommariva E., Pappone C., Martinelli Boneschi F., Di Resta C., Rosaria Carbone M., Salvi E., Vergara P., Sala S., Cusi D., Ferrari M. (2013). Genetics can contribute to the prognosis of Brugada syndrome: A pilot model for risk stratification. Eur. J. Hum. Genet..

[60] Sieira J., Conte G., Ciconte G., Chierchia G.B., Casado-Arroyo R., Baltogiannis G., Di Giovanni G., Saitoh Y., Julia J., Mugnai G. (2017). A score model to predict risk of events in patients with Brugada Syndrome. Eur. Heart J..

[61] Yang Y., Hu D., Sacher F., Kusano K.F., Li X., Barajas-Martinez H., Hocini M., Li Y., Gao Y., Shang H. (2019). Meta-Analysis of Risk Stratification of SCN5A With Brugada Syndrome: Is SCN5A Always a Marker of Low Risk?. Front. Physiol..

[62] Rehm H.L., Bale S.J., Bayrak-Toydemir P., Berg J.S., Brown K.K., Deignan J.L., Friez M.J., Funke B.H., Hegde M.R., Lyon E. (2013). ACMG clinical laboratory standards for next-generation sequencing. Genet. Med..

[63] Chen J.Z., Xie X.D., Wang X.X., Tao M., Shang Y.P., Guo X.G. (2004). Single nucleotide polymorphisms of the SCN5A gene in Han Chinese and their relation with Brugada syndrome. Chin. Med. J..

[64] Poelzing S., Forleo C., Samodell M., Dudash L., Sorrentino S., Anaclerio M., Troccoli R., Iacoviello M., Romito R., Guida P. (2006). SCN5A polymorphism restores trafficking of a Brugada syndrome mutation on a separate gene. Circulation.

[65] Lizotte E., Junttila M.J., Dube M.P., Hong K., Benito B., De Zutter M., Henkens S., Sarkozy A., Huikuri H.V., Towbin J. (2009). Genetic modulation of brugada syndrome by a common polymorphism. J. Cardiovasc. Electrophysiol..

